# Determinants of Body Mass Index Among Vocational College Adolescents

**DOI:** 10.1155/ijpe/2440122

**Published:** 2026-01-22

**Authors:** Galia M. Nasybullina, Svetlana S. Delets, Ekaterina D. Konstantinova, Tatiana A. Maslakova, Anatoly N. Varaksin, Svetlana Yu. Ogorodnikova

**Affiliations:** ^1^ Institute of Preventive Medicine, Ural State Medical University, Ekaterinburg, Russia, usma.ru; ^2^ Department of General Hygiene, South-Ural State Medical University, Chelyabinsk, Russia, chelsma.ru; ^3^ Biostatistics Laboratory, Institute of Industrial Ecology, Ural Branch of the Russian Academy of Sciences, Ekaterinburg, Russia, ras.ru

**Keywords:** adolescents, body mass index, nutrition, physical activity, secondary vocational school students, smoking

## Abstract

The objective of this study was to investigate the prevalence of deviations from normal body weight among students of secondary vocational educational institutions and their association with dietary and nondietary factors. The transition from adolescence to adulthood is a unique period during which lifelong eating habits are formed. The study involved 401 boys and 408 girls aged 15–17 years. Height and weight were measured; a questionnaire on diet, meal frequency, and portion size was completed to assess the set of consumed products, nutritional value of the diet, physical activity level, and smoking status. Descriptive statistics, Student′s *t*‐test, Mann–Whitney test, and multiple linear regression were used. The prevalence of underweight was 16% among boys and 15% among girls; the prevalence of overweight was 11.2% and 13.7%, and the prevalence of obesity was 4.2% and 5.1%, respectively. Overweight/obese young adults had higher median consumption of brown bread, dairy products, and eggs compared with normal weight young adults. Overweight girls ate more meat and potatoes. Our results add to the literature linking the prevalence of underweight among adolescents to dietary and nondietary factors. Our results suggest that improving nutrition among students may reduce the risk of chronic diseases in the future.

## 1. Introduction

The importance of the problem of overweight and obesity in the current context is beyond doubt. According to the Rosstat survey for 2018, 17.8% of men and 24.5% of women had first, second, and third degree obesity. Overweight was recorded in 46.9% of men and 34.7% of women. Only 34% of men and 38.1% of women corresponded to the normal characteristics. Over the past 20 years, the proportion of obese children in the age group of 6–11 year olds increased from 7% to 13%, whereas in the group of 12–19 year olds, from 5% to 14%. The number of those who would find themselves short of food occasionally or often was 4.6%. The survey also revealed differences in food habits between men and women and adults and children [1]. Being obese is associated with various consequences such as declining quality of life, development of emotional and behavioral problems, and worsening socioeconomic circumstances [2].

Meanwhile, there is a critical time for establishing nutrition behaviors carried into adulthood. Foundations for future obesity (or lack of it) are laid during adolescence. The transition between adolescence and adulthood, characterized by increasing independence, autonomy, and responsibility, is often the first time period in which individuals make autonomous decisions about “how, what, where, and when to eat” [3] and is, therefore, a crucial life‐stage for establishing life‐long health behaviors and habits, including healthy eating patterns [4]. For many, the transition into adulthood results in a shift in composition and quality of the diet. In fact, most studies have found that diet quality may worsen during this transition [5].

It should be noted that regional obesity and malnutrition epidemiology studies, in both Russia and other countries, are mostly conducted among either schoolchildren or university students. Meanwhile, there is another large group that remains overlooked: secondary vocational school students (SVSSs), who do display particular characteristics in their health, socioeconomic status, behavioral factors, and education and upbringing conditions [6, 7]. Meanwhile, this stratum requires additional in‐depth research, since they are extremely vulnerable in terms of health risks. SVSSs are very much different from university students culturally, educationally, and financially [8]. SVSSs present a fairly homogeneous group in many respects. Basically, they come from families that are not affluent, do not rank high in social status, and level of parents′ education. Their parents cannot provide their children with healthy food not only because it is expensive but also because they lack knowledge and education. As a rule, their children grow up and reproduce the parental model of lifestyle. It is not the most prosperous and healthy lifestyle, but it is reproduced by people who are of value to society from a humanitarian, social, and economic point of view. Students of secondary vocational schools are learning blue‐collar trades and, respectively, in a couple of years, they will be working in key areas of industrial production. Thus, according to the 2020 census of the Russian Federation, 37.9% of the able‐bodied population of Russia has a secondary vocational education.

According to recent studies, higher mean body mass index (BMI) values are characteristic of children living in developed countries, but developing countries are demonstrating the highest rates of increase in overweight and obesity [9]. At the same time, in many countries, the problem of general protein–energy malnutrition manifesting itself in the form of body weight deficiency presents a pressing issue [10]. This is applicable to Russia as well. Thus, according to the 2018 selective survey of diets in the Russian Federation, the prevalence of overweight in the general population of children aged 2–18 years estimated according to the WHO reference curves amounted to 18%, that of obesity amounted to 9.1%, and the index of malnutrition as the sum of underweight, stunting, wasting rates, and their combination was 8.0% [11].

Despite a large number of studies on overweight and obesity epidemiology, risk factors, and malnutrition, there are controversial issues remaining.

First, are there any specifics in the impact of risk factors on various subpopulations in the child population, such as the region of residence, the level of urbanization, and the level of education? Understanding these specifics is important for differentiated targeting of preventive actions and for increasing their effectiveness. Second, what methods of analysis should be used to identify causal relationships between deviations in health and risk factors in studies?

Researchers generally agree that underweight, overweight, and obesity are the result of a whole range of influencing factors, among which nutritional factors are given priority, in particular imbalance in energy consumption and expenditure [12, 13]. Moreover, just as important are factors such as a regular meal pattern, dietary choices [14, 15], and the contribution of individual nutrients to the calorie content of the diet [16–18].

Somewhat less often studies are concerned with nonfood factors, such as inherited predisposition, family food traditions, socioeconomic factors, food and ready‐meal preferences, hygienic literacy, and availability and quality of nutrition education services [19, 20].

Some studies fail to find seemingly obvious associations between BMI, on the one hand, and such factors as calorie intake or physical activity levels on the other [18, 21]. This could be due to a large number of parameters employed to characterize nutritional and nonnutritional risk factors and the relationships between them since factors can correlate with each other. Therefore, there is an ongoing search for ways to integrate nutrition characteristics into a shorter list of indicators, on the one hand, and to find multivariate analysis methods to deal with heterogeneous and often poorly formalized information on the other [22].

Regarding the shortlisting mentioned above, it was found, for instance, that increased BMI in adolescents is associated with a western diet characterized by high protein and fat content [5, 17, 23]. The use of multivariate analysis methods (for example, different variants of regression analysis) makes it possible to identify the most significant predictors among them without preliminary consolidation of information about risk factors [24–27].

Researchers also commonly focus on gender differences in underweight, overweight, and obesity rates and dietary habits, failing to explore the role of gender differences when assessing the effect of certain factors on health problems studied.

The main gaps that our research fills are as follows:

1. Focus on an understudied population: SVSSs

• Gap: The majority of epidemiological studies concerning obesity and malnutrition, both in Russia and internationally, focus predominantly on either general school students or university students.
• Our contribution: We specifically investigate SVSSs. We reasonably argue that this group possesses unique characteristics (including socioeconomic status, living conditions, and cultural/educational differences compared with university students) that render them vulnerable and necessitate separate investigation.

2. A comprehensive investigation of BMI‐related factors (including undernutrition)

While the problem of overweight is extensively studied, our research offers a more complete set of analyzed factors specifically for this population group:

• Gap: There is a lack of data detailing precisely which dietary and nondietary factors influence the entire spectrum of deviations from normal weight (not only obesity but also underweight) specifically within this age group (15–17 years old).
• Our contribution: We investigate not only the prevalence of overweight/obesity (11.2% and 5.1%, respectively) but also undernutrition (16% among boys and 15% among girls), which is a crucial aspect of adolescent health. We correlate these findings with specific dietary components (consumption of dark bread, dairy products, eggs, meat, and potatoes) and nondietary factors (physical activity, smoking).

3. Focus on a critical period for habit formation

We emphasize that the transition from adolescence to adulthood is a critical juncture for the establishment of lifelong habits:

• Gap: There is a scarcity of research linking lifestyle factors during adolescence (before entering higher education or achieving full independence) to future risks of chronic diseases.
• Our contribution: Our study focuses specifically on how eating patterns are formed during this precise period (ages 15–17 in vocational secondary education institutions [VSEIs]), patterns that may lead to adverse outcomes later in life.

Thus, our research fills this gap by providing essential epidemiological data and risk factors for an understudied and vulnerable group, vocational secondary school students, during a critical phase of their development.

The objective of the study is to investigate the prevalence of abnormal BMI categories (underweight, overweight, and obesity) among students aged 15–17 attending secondary vocational education institutions and identify associated dietary and nondietary risk factors. To meet this objective, a cross‐sectional survey was conducted involving 809 students (401 male and 408 female). Anthropometric measurements (height and weight) were recorded. Data on dietary habits (meal frequency, portion sizes, and food consumption patterns), physical activity levels, and smoking status were collected through standardized questionnaires. The collected data were analyzed using descriptive statistics, Student′s *t*‐test, Mann–Whitney *U* test, and multiple linear regression models.

## 2. Material and Methods

### 2.1. Study Design

This study utilized a cross‐sectional design and forms a component of a larger epidemiological study focusing on the health and nutrition status of adolescents attending secondary vocational educational institutions (SVEIs) in Chelyabinsk, a major industrial center in the Russian Federation with a population exceeding one million (a prior analysis examining nutrition and BMI association based on smoking status, using the same cohort, has been previously published).

The study protocol received approval from the Local Ethics Committee of the South Ural State Medical University (Minutes No. 11, dated 9 November 2013).

The study sample comprised students from four selected colleges in Chelyabinsk, chosen to represent diverse vocational training areas, including metallurgy, construction, transport, textile industry, education, law, and art. Collectively, these four institutions enroll approximately 75% of all students pursuing secondary vocational education in Chelyabinsk.

The target population for this analysis included all students aged 15–17 years enrolled in these colleges who provided voluntary informed consent to participate. Exclusion criteria were established to minimize confounding factors related to nutrition: adolescents with chronic diseases requiring specific medical or dietary nutrition, those with acute illnesses within 14 days prior to the survey, and pregnant or lactating women

Initially, 888 students were referred to the medical office of the secondary vocational education institutions. Of these, 15 adolescents declined participation immediately. Furthermore, 5 individuals were excluded for not meeting inclusion criteria (e.g., being vegetarian, on a therapeutic diet, or pregnant), 10 respondents provided unreliable information, and 49 were unable to complete all necessary details. The final study sample consisted of 809 participants (401 boys and 408 girls).

### 2.2. Research Methods

Height and body weight were measured by anthropometry in the medical rooms of the colleges, and the measurement results were used to calculate the BMI (kg/m^2^). The indicators were assessed using the WHO growth reference for schoolchildren and adolescents aged 5–19 years [28]. Each adolescent was assigned to one of the five weight groups: severely underweight (BMI‐for‐age < 3rd percentile), underweight (BMI 3rd–15th percentile), normal body weight (BMI 15th–85th percentile), overweight (BMI 85th–97th percentile), and severely overweight or obese (BMI > 97th percentile) [29]. The body fat indicators determined by measuring abdominal skinfold thickness and waist circumference point to the abdominal type of fat deposition. The criteria of excess fat deposition were a skinfold thickness of more than 10 mm [30] and a waist circumference of 80 cm or more for girls and 94 cm or more for boys [31].

The sociodemographic indicators, nutrition, physical activity, and smoking were studied by interviewing the participants. The sociodemographic indicators included the parents′ level of education (higher, secondary vocational, secondary general, or incomplete secondary), family affluence self‐assessment (lower than others, the same as others, or higher than others), and housing conditions (a separate apartment with conveniences, a detached house with conveniences, a detached house without conveniences, a hostel, or a room in a shared communal apartment). Also, the survey established who the adolescents were living with (parents, other relatives, a male or female friend, and alone).

Nutrition was studied by finding out the frequency of food consumption during the last 30 days with quantitative (in grams or milliliters) estimation of the actual consumption of various food groups; the list included 67 foods [32]. To determine the amount of food intake, we used an album of food amounts and dish portions [33]. For each food, we determined consumption frequency using the following variants: did not consume, 1–2 times a month, 3–4 times a month, 2–3 times a week, 4–6 times a week, 1–2 times a day, 3–4 times a day, or 5 or more times a day.

Based on the results of the survey, we calculated the consumption of basic foods in conditional servings and grams, the total amount of food consumed on average per day, and the nutritional value of the diet: calories, proteins, fats (including cholesterol, saturated, monounsaturated, and polyunsaturated fatty acids), carbohydrates (including simple sugars, polysaccharides, and dietary fiber), vitamins A, B1, B2, PP, C, and E, calcium, iron, magnesium, sodium, phosphorus, potassium, the proteins–fats–carbohydrates ratio by weight, and the contribution of proteins, fats, and carbohydrates to the diet calories as a percentage.

In addition, the questionnaire included questions about adolescents′ diets (number of meals during school days and weekends), meal taking at college, participation in meal cooking at home, self‐reported appetite and food intake, and adolescent parents′ height and body weight and joint home meal cooking.

To determine the level of physical activity, we studied the usual duration of the main activities during the day in hours: sleep, sedentary or standing‐posture activities, moderate physical activities and walking, and high‐level physical activities (sports, dancing, etc.), followed by a calculation of the daily energy expenditure and physical activity coefficient (PAC) [34].

The students were asked three questions about smoking: whether they smoke at all (yes or no), at what age they started smoking, and how many cigarettes they smoke daily [35]. For adolescent smokers, we calculated the smoking index (SI) using a well‐known formula:

SI=pack‐year=number of cigarettes smoked per day·years of tobacco use 20.

### 2.3. Statistical Analysis

To identify nutritional and nonnutritional risk factors, the adolescents were divided into three comparison groups depending on their BMI: underweight (BMI‐for‐age index < 15th percentile), normal body weight (BMI‐for‐age index 15th–85th percentile), and overweight (BMI‐for‐age index > 85th percentile). Given the low prevalence of participants falling into the severely underweight (e.g., BMI‐for‐age index < 5th percentile) and severely obese (e.g., BMI‐for‐age index > 95th percentile) categories within this cohort, these participants were integrated into the broader underweight (BMI‐for‐age index < 15th percentile) and overweight (BMI‐for‐age index > 85th percentile) groups, respectively. This decision was made to ensure adequate statistical power for the primary group comparisons. For descriptive statistics, we calculated extensive indicators for qualitative features and medians and interquartile ranges for quantitative characteristics. To assess the statistical significance of differences, Student′s and Mann–Whitney tests were performed. The analysis was carried out separately for boys and girls.

Statistica for Windows 10 was used for data analysis. The statistical significance of differences was estimated at the significance level *α* = 0.05.

### 2.4. Regression Analysis

To establish the structure of the relationship between BMI and indicators characterizing the nutrition of adolescents and nonnutritional factors (physical activity, smoking, and some family factors), a multivariate regression analysis was carried out. The latter was performed using the moving average technique to reduce the variance of the indicators and reveal the structure of the relationship between BMI and indicators characterizing adolescent nutrition and nonnutritional factors in regression models [36]. Next, we applied a stepwise linear regression procedure.

Multiple linear regression models were constructed separately for boys and girls (line 200). This stratification was necessary due to known sex‐specific differences in physiological maturation, body composition trajectories, and the differential impact of nutritional status on the measured outcomes in pediatric populations [37].

## 3. Results

### 3.1. Sample Characteristics

**Figure 1:**
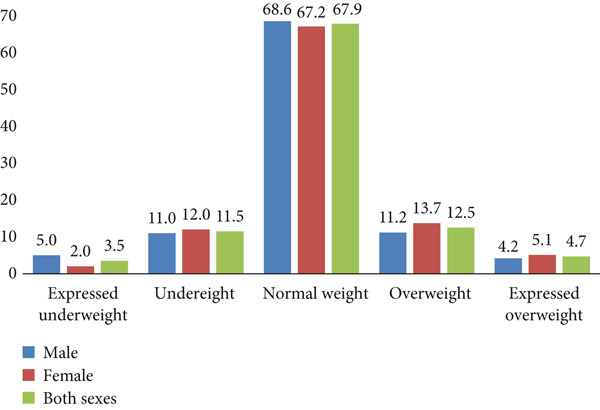
Distribution of boys and girls by BMI categories, percentage.

The distribution of boys and girls by body weight was close to the WHO reference population (Figure 1).

No statistically significant differences were found in overweight plus obesity rates and underweight rates between girls and boys. Stunting (height‐for‐age index < 3rd percentile) was more frequent among boys (5.5% vs. 1.5% among girls; *p* < 0.05).

The indicators of waist circumference and abdominal skinfold thickness in adolescents with different BMI values are presented in Table 1.

**Table 1:** Waist circumference and abdominal skinfold thickness in adolescents with different BMI values, median (25th and 75th percentiles of median).

**Indicator**	**Male**			**Female**		
	**Underweight** **n** = 64	**Normal weight** **n** = 275	**Overweight** **n** = 62	**Underweight** **n** = 57	**Normal weight** **n** = 274	**Overweight** **n** = 77
Waist circumference, cm	67∗ (63–70)	72 (69–74)	84∗ (80–88)	60∗ (58–64)	65 (63–69)	76∗ (72–83)
Thickness of the skin fold in the abdomen, mm	0.5∗ (0.0–2.0)	4.0 (2.0–8.0)	17.0∗ (12.0–24.0)	1.0∗ (0.0–2.0)	6.0 (2.0–8.0)	15.0∗ (12.0–20.0)

^∗^ *p* < 0.05 compared with the normal body weight group.

The median values of waist circumference and abdominal skinfold thickness were the lowest among underweight participants and the highest among overweight and obese individuals. There were no adolescents with signs of excessive fat deposition in the underweight group. Among those with normal body weight, waist circumference exceeded the permissible values in isolated cases (one boy and three girls), whereas skinfold thickness was excessive in 17.1% of boys and 21.5% of girls. In the overweight or obesity group, signs of excessive fat deposition were found in 13% of boys and 34% of girls in terms of waist circumference and in 79% of boys and 92.2% of girls in terms of skinfold thickness (*p* < 0.05).

### 3.2. Nutrition Characteristics

The distribution of adolescents with different BMI values by meal‐taking frequency on school days and weekends is presented in Table 2. Our comparison group is the normal body weight group.

**Table 2:** Distribution of adolescents with different BMI values by meal‐taking frequency on school days and weekends, abs (%).

**Frequency of food intake during the day**	**Male**			**Female**		
	**Underweight** **n** = 64	**Normal weight** **n** = 275	**Overweight** **n** = 62	**Underweight** **n** = 57	**Normal weight** **n** = 275	**Overweight** **n** = 62
During study days						
1–2	13 (20.3%)	46 (16.7%)	18∗ (29.0%)	16 (28.1%)	76 (27.7%)	21 (27.3%)
3	36 (56.3%)	129 (46.9%)	30 (48.4%)	28 (49.1%)	122 (44.5%)	40 (51.9%)
4	12 (18.8%)	70 (25.5%)	11 (17.7%)	9 (15.8%)	58 (21.2%)	13 (16.9%)
5 times or more	3 (4.7%)	30 (10.9%)	3 (4.8%)	4 (7.0%)	18 (6.6%)	3 (3.9%)
On weekends						
1–2	8 (12.5%)	32 (11.6%)	10 (16.1%)	8 (14.0%)	43 (15.7%)	6 (7.8%)
3	28 (43.8%)	113 (41.1%)	32 (51.6%)	25 (43.9%)	103 (37.6%)	31 (40.3%)
4	23 (35.9%)	77 (28.0%)	12 (19.4%)	18 (31.6%)	86 (31.4%)	27 (35.1%)
5 times or more	5∗ (7.8%)	53 (19.3%)	8 (12.9%)	6 (10.5%)	42 (15.3%)	13 (16.9%)

∗*p* < 0.05 compared with the normal body weight group.

Eating problems in the form of rare meal taking were more often found among overweight young men. Overweight girls, on the contrary, tended to eat more often than their normal body weight or underweight peers, but only on weekends. On school days, overweight girls were less likely to respond that they ate daily in the college canteen (29.9%), whereas the proportion of such respondents was 44.2% among those with normal body weight and 56.1% (*p* < 0.05) among underweight adolescents.

Table 3 shows the consumption of the main groups of foods expressed in conventional servings. Our comparison group is the normal body weight group.

**Table 3:** Consumption of the main groups of foods and dishes with daily diets among adolescents with different BMI values, conventional servings per day, median (25th and 75th percentiles of median).

**Products and dishes**	**Recommended by WHO**	**Sex**	**Underweight**	**Normal weight**	**Overweight**
Bread products, cereals, and pasta dishes	6.0–11.0	Male	10.5 (7.0–15.6)	11.5 (7.0–16.6)	11.8 (7.4–14.7)
		Female	7.5∗ (4.7–11.5)	6.1 (3.6–9.6)	6.5 (3.9–10.2)
Meat products, fish products, and dishes from them	2.0–3.0	Male	3.8 (1.6–6.6)	3.9 (2.4–6.3)	4.5 (3.1–6.3)
		Female	2.8 (1.7–4.4)	3.1 (1.8–4.4)	2.8 (1.7–3.8)
Dairy products and meals	2.0–3.0	Male	1.7 (0.8–3.5)	2.3 (1.0–4.5)	4.4 (2.5–6.1)
		Female	2.2 (1.1–3.7)	2.4 (1.2–3.9)	3.2 (1.0–4.8)
Egg dishes	0.5	Male	0.4 (0.1–0.7)	0.2 (0.1–0.7)	0.4 (0.1–1.1)
		Female	0.1 (0.1–0.4)	0.1 (0.0–0.4)	0.2 (0.1–0.7)
Sugar and confectionery	1.0	Male	9.5 (4.9–13.6)	8.2 (4.3–12.6)	8.1 (4.8–10.5)
		Female	6.0 (2.8–9.9)	6.0 (3.2–9.9)	6.6 (3.4–11.6)
Vegetable dishes	3.0–5.0	Male	3.6 (2.0–5.0)	3.4 (2.1–5.2)	3.7 (2.3–5.0)
		Female	2.5 (1.6–3.6)	2.7 (1.8–4.3)	2.5 (1.7–4.1)
Fruits, berries, and dishes from them	2.0–4.0	Male	0.9 (0.5–1.8)	1.0 (0.5–1.8)	0.8 (0.5–1.8)
		Female	0.9 (0.5–1.7)	1.1 (0.4–1.8)	0.8 (0.4–1.4)
Oils and fats	2.0–3.0	Male	1.8 (0.9–3.0)	2.0 (0.8–3.5)	1.7 (0.8–4.0)
		Female	1.3 (0.4–2.6)	1.5 (0.6–2.9)	1.4 (0.7–2.6)

^∗^ *p* <0.05 compared with the normal body weight group, recommended by WHO [38].

The consumption of the main groups of foods expressed in conventional servings differed significantly between boys and girls in general. Compared with girls, boys were more likely to eat almost all groups of foods with the exception of dairy products, fruits, and berries, and statistically significant differences between individuals with different BMI values were found only for one group of foods: bakery, cereals, pasta, and potatoes. These were more often consumed by underweight girls.

Table 4 presents the consumption of the main groups of foods per day in grams by adolescents with different BMI values. Our comparison group is the normal body weight group.

**Table 4:** Consumption of the main foods by adolescents with different BMI values, median (25th and 75th percentiles of median), grams per day.

**Food**	**Recommended quantity**	**Sex**	**Underweight**	**Normal weight**	**Overweight**
Bakery products, incl.	360	Male	252.9 (182.8–390.5)	291.0 (157.0–399.1)	275.7 (200.2–369.2)
		Female	162.6 (98.2–274.2)	142.3 (77.9–226.1)	141.9 (82.7–236.3)
Rye bread	120	Male	28.6 (0.0–120.0)	28.5 (0.0–120.0)	60.0∗ (2.0–180.0)
		Female	14.0 (0.0–60.0)	14.3 (0.0–60.0)	28.5 (2.0–60.0)
Wheat bread	200	Male	81.3 (17.8–175.0)	89.1 (35.6–175.0)	82.1 (26.7–150.0)
		Female	35.6 (5.9–75.0)	17.8 (1.3–75.0)	26.8 (2.9–75.0)
Cereals, legumes	50	Male	67.2 (19.2–143.8)	77.0 (7.5–157.1)	127.6 (47.8–254.2)
		Female	82.8 (30.1–142.8)	70.3 (20.0–138.7)	82.8 (19.9–145.1)
Pasta	20	Male	53.5 (17.6–106.9)	53.5 (17.6–106.9)	53.5 (17.5–106.9)
		Female	53.5 (17.6–106.9)	17.6 (15.0–53.5)	53.5 (17.6–53.5)
Potato	187	Male	78.9 (30.9–124.6)	65.3 (29.3–124.9)	74.5 (30.9–124.6)
		Female	35.7 (19.2–88.9)	34.2 (15.0–78.9)	43.2∗ (23.4–107.1)
Vegetables (fresh, frozen, canned)	320	Male	297.3 (163.0–419.6)	272.4 (148.4–460.0)	315.6 (199.3–478.9)
		Female	231.7 (97.8–351.0)	245.5 (140.4–405.4)	220.0 (137.2–374.5)
Fresh fruits	185	Male	131.8 (74.2–270.6)	144.8 (67.5–267.8)	121.7 (71.1–267.7)
		Female	138.7 (68.0–248.4)	158.3 (61.0–264.1)	114.4 (61.0–213.9)
Fruit juices, fortified drinks	200	Male	118.2 (69.1–214.2)	142.8 (56.8–285.6)	71.4∗ (23.4–142.8)
		Female	46.8 (20.0–94.8)	71.4 (23.4–152.6)	71.4 (20.0–142.8)
Animal and poultry meat, including by‐products	171	Male	120.8 (52.7–206.0)	132.6 (76.5–228.5)	147.4 (82.1–228.0)
		Female	76.1 (43.5–132.1)	90.3 (44.3–147.6)	123.8∗ (75.0–163.7)
Sausages	Not standardized	Male	49.7 (20.0–114.1)	58.5 (21.8–110.7)	58.0 (18.7–103.5)
		Female	22.2 (5.0–99.8)	28.2 (10.5–60.7)	36.7 (11.7–80.0)
Fish (fillet), incl. fillet lightly or lightly salted	77	Male	6.5 (0.0–21.4)	7.0 (0.0–18.7)	10.7 (3.5–30.0)
		Female	4.0 (0.0–11.0)	7.0 (1.5–15.0)	12.4 (0.0–21.7)
Milk	350	Male	59.1 (10.0–142.8)	71.4 (10.0–285.6)	142.6∗ (46.8–300.0)
		Female	71.4 (4.7–285.6)	71.4 (0.0–285.2)	46.8 (10.0–142.8)
Sour milk drinks	180	Male	10.0 (0.0–71.4)	23.4 (0.0–71.4)	71.4∗ (0.0–142.8)
		Female	71.4 (0.0–142.6)	23.4 (0.0–142.6)	23.4 (0.0–71.4)
Curd (cottage cheese)	60	Male	0.0 (0.0–6.1)	1.7 (0.0–11.7)	5.0∗ (0.0–24.2)
		Female	2.0 (0.0–18.2)	4.1 (0.0–12.1)	2.0 (0.0–15.8)
Cheese	15	Male	7.1 (0.5–21.4)	4.7 (0.0–21.4)	14.2∗ (2.0–30.0)
		Female	7.0 (1.0–21.4)	7.1 (2.0–21.4)	4.7 (2.0–21.4)
Sour cream	10	Male	2.1 (0.0–12.8)	2.1 (0.0–12.9)	6.3∗ (0.0–19.2)
		Female	2.1 (0.0–6.4)	2.1 (0.0–6.4)	1.8 (0.0–6.4)
Butter	35	Male	1.2 (0.0–7.1)	1.2 (0.0–7.1)	0.5 (0.0–3.5)
		Female	0.5 (0.0–3.6)	1.2 (0.0–7.1)	1.0 (0.0–7.1)
Vegetable oil	18	Male	3.0 (0.0–12.1)	6.1 (0.0–12.1)	6.1 (0.9–12.1)
		Female	4.0 (0.9–24.2)	6.1 (0.9–12.1)	6.1 (0.9–24.2)
Mayonnaise	Not standardized	Male	5.4 (1.3–22.5)	10.7 (1.8–22.5)	10.7 (1.8–22.5)
		Female	3.5 (0.0–10.7)	5.4 (0.8–21.4)	5.4 (0.8–10.7)
Egg	45	Male	13.2 (2.3–32.1)	10.5 (2.3–32.1)	24.1∗ (5.3–48.2)
		Female	5.3 (0.0–16.1)	5.3 (2.3–16.1)	5.3 (0.0–16.1)
Sugar (including for cooking and drinks)	35	Male	31.5 (10.5–70.0)	31.5 (10.5–70.0)	33.3 (21.0–49.0)
		Female	21.0 (0.0–31.5)	12.7 (0.0–31.5)	21.0 (1.6–49.0)
Confectionery	15	Male	45.2 (21.2–75.0)	38.0 (15.5–73.1)	28.2 (13.0–56.4)
		Female	54.0 (18.0–79.2)	47.9 (22.0–73.9)	45.4 (19.7–77.0)
Tea (g)	2	Male	3.6 (3.0–7.0)	7.0 (3.0–7.0)	7.0 (3.0–7.0)
		Female	7.0 (3.0–7.0)	6.0 (3.0–7.0)	7.0 (3.0–7.0)
Coffee drink (g)	2	Male	2.4 (0.0–11.1)	2.3 (0.0–15.0)	3.5 (0.0–15.0)
		Female	1.2 (0.0–7.1)	1.2 (0.0–7.1)	1.2 (0.0–3.6)

^∗^ *p* < 0.05 compared with the normal body weight group for children aged 12 and over [39].

Differences in the amount of food consumed in the comparison groups appeared as trends. The amount of consumed food by median values was the lowest among underweight young men at 2569.4 g (1927.1–3155.7) per day and the highest among overweight boys 2936.7 g (2158.4–3446.5) per day (*p* = 0.097). For girls, the amount of consumed food between the comparison groups did not differ statistically significantly, ranging from 1973.6 (1638.8–2315.5) in the underweight group to 2151.57 g (1825.3–2889.4) per day in the overweight group (*p* > 0.05). Regarding the consumption of individual foods, statistically significant differences between the comparison groups were found mainly for young men (Table 4); overweight young men tended to consume more brown bread, dairy products (with the exception of milk itself), and eggs, and they consumed less juices and juice‐containing drinks than the others did. Overweight girls differed from underweight or normal body weight girls in terms of a higher consumption of potatoes and meat.

The nutritional value of the daily diets consumed by adolescents with different BMI values is presented in Table 5. Our comparison group is the normal body weight group.

**Table 5:** Nutritional value of daily diets for adolescents with different BMI values, median (25th and 75th percentiles of median).

**Nutrient substance, units of measurement**	**Sex**	**Norms of physiological needs**	**Underweight**	**Normal weight**	**Overweight**
Diet calories, kcal	Male	2900	3098.9 (2193.0–3877.5)	3022.6 (2313.2–3898.4)	3291.2 (2653.7–3939.5)
	Female	2500	2261.3 (1848.1–2961.6)	2244.9 (1853.8–2774.7)	2496.1∗ (2000.5–3082.1)
Proteins, g	Male	87	85.6 (62.0–119.9)	87.4 (64.1–123.9)	100.8∗ (86.0–128.1)
	Female	75	64.6 (53.3–85.3)	66.3 (52.8–84.5)	75.7∗ (59.2–95.6)
Proteins, % of calories	Male	12–15	11.4 (10.1–13.6)	11.8 (10.2–13.4)	12.9∗ (11.3–14.5)
	Female	12–15	11.7 (10.2–12.9)	11.8 (9.9–13.7)	12.3 (10.9–13.7)
Of which proteins of animal origin, g	Male	—	43.6 (28.2–73.5)	49.7 (31.1–75.0)	63.0∗ (47.5–89.5)
	Female	—	36.5 (24.1–49.1)	38.7 (26.7–55.2)	49.7∗ (36.8–68.6)
Proteins of animal origin, % of proteins	Male	60	54.9 (42.6–64.5)	57.8 (45.1–67.6)	63.1∗ (50.7–71.5)
	Female	60	57.8 (46.0–66.6)	60.7 (47.8–70.7)	65.1∗ (55.0–73.1)
Fats, g	Male	97	109.9 (74.1–163.6)	113.4 (79.3–166.8)	128.6 (104.2–169.2)
	Female	83	89.5 (62.9–120.7)	90.3 (64.9–121.1)	102.5 (70.8–131.3)
Fats, % of calories	Male	25–35	33.6 (28.9–40.6)	35.1 (28.7–41.7)	37.1 (29.6–41.6)
	Female	25–35	37.0 (31.4–43.4)	37.9 (29.9–43.3)	36.9 (32.5–41.5)
Saturated fatty acids, g	Male	—	31.4 (21.3–50.8)	34.2 (23.2–49.6)	41.5∗ (31.2–51.7)
	Female	—	24.5 (19.0–39.1)	26.4 (19.9–35.4)	30.9 (22.4–38.5)
Saturated fatty acids, % of calories	Male	< 10	10.3 (8.4–12.9)	10.6 (8.4–13.1)	11.4 (9.6–13.4)
	Female	< 10	10.9 (8.9–12.8)	11.0 (9.0–13.5)	11.1 (9.2–13.4)
Monounsaturated fatty acids, g	Male	—	39.1 (26.9–63.7)	44.1 (29.7–61.9)	48.7 (37.3–60.9)
	Female	—	32.7 (25.1–47.5)	33.8 (25.0–45.8)	38.6 (26.7–50.5)
Monounsaturated fatty acids, % of calories	Male	< 10	13.1 (10.4–16.1)	13.3 (10.7–15.7)	13.8 (11.8–15.5)
	Female	< 10	14.0 (10.4–16.7)	14.4 (11.4–16.8)	14.0 (12.2–16.1)
Polyunsaturated fatty acids, g	Male	—	21.6 (15.1–35.0)	25.5 (16.6–36.0)	26.9 (15.5–38.5)
	Female	—	19.1 (13.9–31.1)	19.4 (12.7–29.8)	22.2 (13.8–31.8)
Polyunsaturated fatty acids, % of calories	Male	6–10	6.8 (5.5–8.8)	7.4 (5.6–9.4)	6.7 (5.3–9.5)
	Female	6–10	7.8 (5.4–11.6)	7.5 (5.8–10.4)	7.9 (6.4–9.8)
Cholesterol, g	Male	< 0.30	0.50 (0.31–0.75)	0.47 (0.25–0.79)	0.73∗ (0.34–1.16)
	Female	< 0.30	0.39 (0.23–0.66)	0.38 (0.21–0.62)	0.46 (0.21–0.80)
Carbohydrates, g	Male	421	424.0 (264.5–494.4)	402.2 (292.2–495.9)	404.8 (324.1–463.9)
	Female	363	300.9 (222.2–361.9)	278.0 (214.1–361.1)	308.6 (241.8–389.5)
Carbohydrates, % of calories	Male	55–60	52.4 (45.9–59.6)	52.1 (44.9–59.0)	50.0 (43.1–56.8)
	Female	55–60	50.8 (42.7–58.7)	49.3 (43.4–58.8)	50.7 (44.8–55.3)
Polysaccharides, g	Male	—	172.6 (126.2–218.7)	181.7 (120.3–235.9)	191.4 (134.3–231.8)
	Female	—	127.1∗ (89.6–168.3)	101.5 (70.8–153.1)	107.7 (82.0–166.7)
Simple sugars, g	Male	—	237.5 (153.2–288.7)	214.5 (156.9–273.3)	217.4 (159.7–251.4)
	Female	—	160.0 (129.6–213.0)	173.0 (131.8–224.8)	186.1∗ (150.6–240.6)
Simple sugars, % of calories	Male	< 10	28.5 (24.1–33.2)	28.1 (23.4–32.8)	27.0 (22.3–29.9)
	Female	< 10	28.9 (22.2–34.1)	30.3 (24.8–36.0)	31.5 (26.0–36.3)
Dietary fiber, g	Male	22	23.9 (16.1–30.5)	24.2 (17.4–32.0)	23.7 (18.1–30.8)
	Female	18	17.3 (12.5–23.6)	16.1 (11.3–24.0)	15.3 (11.1–23.4)
P:F	Male	1:1	1:1.3 (1:1.1–1:1.4)	1:1.3 (1:1.1–1:1.5)	1:1.2 (1:1.0–1:1.5)
	Female	1:1	1:1.3 (1:1.1–1:1.6)	1:1.3 (1:1.2–1:1.6)	1:1.3 (1:1.2–1:1.5)
P:C	Male	1:4	1:4.5 (1:3.4–1:5.9)	1:4.38 (1:3.48–1:5.54)	1:4.13 (1:2.94–1:4.64)
	Female	1:4	1:4.17 (1:3.42–1:5.69)	1:4.25 (1:3.17–1:5.48)	1:4.17 (1:3.34–1:4.91)
Vitamin A (retinol), mcg ret. equiv.	Male	1000	523.1 (326.7–1082.0)	690.4 (326.1–1401.8)	899.8∗ (550.8–1219.6)
	Female	800	352.0 (250.8–733.7)	443.5 (265.7–827.6)	646.4 (286.0–1028.4)
Vitamin B1 (thiamin), mg	Male	1.5	1.3 (0.9–1.8)	1.3 (1.0–1.7)	1.5 (1.2–1.8)
	Female	1.3	1.0 (0.8–1.3)	0.9 (0.7–1.2)	1.1 (0.8–1.3)
Vitamin B2 (riboflavin), mg	Male	1.8	3.7 (1.7–12.0)	3.6 (1.9–8.5)	6.5∗ (2.6–10.1)
	Female	1.5	1.4 (0.9–1.9)	1.3 (1.0–2.0)	1.6 (1.0–2.5)
Vitamin PP (niacin), mg niac. equiv.	Male	20	15.3 (11.7–23.0)	16.4 (12.1–22.6)	17.8 (14.9–22.4)
	Female	18	10.5 (8.6–14.8)	12.4 (8.9–15.7)	14.9 (11.2–17.1)
Vitamin C (ascorbic acid), mg	Male	90	103.6 (75.3–154.6)	111.7 (71.7–176.2)	112.7 (79.1–172.2)
	Female	70	82.4 (52.2–138.1)	96.5 (61.1–151.8)	87.2 (59.8–141.8)
Vitamin E, mg current equivalent	Male	15	19.0 (14.7–29.7)	22.4 (15.0–31.1)	23.5 (14.5–33.9)
	Female	15	16.8 (11.7–21.9)	15.7 (10.4–23.4)	17.8 (12.1–25.1)
Calcium, mg	Male	1200	766.7 (546.6–1025.0)	826.7 (563.8–1202.9)	1162.2∗ (883.7–1536.4)
	Female	1200	740.1 (487.4–950.5)	686.8 (505.0–1025.2)	778.4 (531.5–1181.0)
Iron, mg	Male	15	20.5 (14.8–26.6)	20.6 (14.9–26.1)	21.2 (16.8–28.9)
	Female	18	14.4 (11.7–18.4)	15.3 (11.6–20.2)	15.9 (12.1–19.38)
Magnesium, mg	Male	400	346.7 (237.6–448.8)	350.8 (261.2–442.6)	382.6∗ (308.4–472.0)
	Female	400	252.1 (207.9–304.6)	254.5 (196.6–328.3)	278.5 (220.4–354.8)
Phosphorus, mg	Male	900	1418.6 (950.4–1775.0)	1344.5 (1014.0–1842.4)	1697.2∗ (1314.4–2010.5)
	Female	900	1061.7 (889.2–1296.8)	1085.2 (839.4–1344.9)	1199.9∗ (838.8–1522.2)
Sodium, mg	Male	1300	4532.1 (2666.2–6501.5)	4314.6 (3336.7–6058.6)	4788.9∗ (3994.1–5987.5)
	Female	1300	3100.6 (2517.7–4173.2)	3057.5 (2296.9–4190.6)	3515.4∗ (2569.0–4493.2)
Potassium, mg	Male	3200	3539.0 (2596.1–4287.3)	3515.4 (2615.9–4482.4)	3989.4 (2750.8–4816.6)
	Female	3200	2601.3 (2105.2–3326.5)	2760.2 (2100.5–3634.9)	2844.3 (2191.2–3638.6)
Calcium:phosphorus ratio	Male	1:1.5	1:1.82 (1:1.48–1:2.10)	1:1.69 (1:1.41–1:2.02)	1:1.48 (1:1.23–1:1.78)
	Female	1:1.5	1:1.46 (1:1.26–1:1.82)	1:1.48 (1:1.25–1:1.80)	1:1.54 (1:1.24–1:1.83)
Calcium:magnesium ratio	Male	1:0.3	1:0.48 (1:0.34–1:0.57)	1:0.43 (1:0.33–1:0.55)	1:0.34 (1:0.27–1:0.46)
	Female	1:0.3	1:0.38 (1:0.28–0.51)	1:0.36 (1:0.27–1:0.49)	1:0.35 (1:0.27–1:0.48)

^∗^ *p* < 0.05 compared with the normal body weight group for boys and girls aged 15–17 [40].

In terms of nutritional value of the diets, differences were found for overweight or obese adolescents. The median calorie values for overweight girls exceeded those in the comparison groups. Among young men, no statistically significant differences were revealed in caloric contents between the comparison groups. Both overweight girls and overweight boys tended to consume more proteins with food, including animal proteins. Their diets were also characterized by the highest median values of protein contribution to the calorie content and the proportion of animal proteins in the total protein content in their diets. As for fat and carbohydrate contents, the comparison groups did not differ statistically significantly for both boys and girls. However, overweight boys tended to have the highest median intakes of saturated fatty acids and cholesterol. Overweight girls consumed higher amounts of simple sugars and less polysaccharides. Individual vitamins and minerals were also found to be higher in content in the diets of overweight adolescents, which can be attributed to the high consumption of foods that are the source of these micronutrients.

Differences were found in several indicators that affect the eating behavior of adolescents. Underweight boys were less likely to report good appetite than those with normal weight or overweight (40.6% vs. 67.6% and 59.7%, respectively). Underweight or normal weight boys tended to report more often that they ate only what they liked (42.2% and 41.5%, respectively), with only 29.0% (*р* = 0.055) of overweight boys reporting the same.

### 3.3. Nonnutritional Characteristics

A significant proportion of the adolescents did not know how to correctly estimate their weight. 34.4% of boys and 50.9% of girls in the underweight group, and 56.5% of boys and 22.1% of girls in the overweight group considered their weight to be normal. Among adolescents with normal body weight, 65.8% of boys and 70.4% of girls believed that their weight corresponded to the norm. At the same time, 25.5% of young men with normal body weight considered their weight to be insufficient, whereas the same number of girls regarded themselves to be overweight.

In terms of physical activity level, the comparison groups did not differ statistically significantly for both boys and girls. Young men with underweight or normal body weight had almost the same median values of the PAC: 2.0 (1.8–2.3) and 2.0 (1.8–2.2), respectively, whereas overweight men had a PAC of 1.9 (1.9–2.1). Among girls, the median PAC values in the same groups were 1.9 (1.7–2.0), 1.8 (1.6–2.0), and 1.7 (1.6–2.0). These PAC values characterize the average level of physical activity in the study sample [41].

The proportion of smokers was the highest among young men with underweight or normal weight (48.4% and 41.5%, respectively), whereas among the overweight it is significantly lower at 25.8% (*p* < 0.05). Among girls, the prevalence of smoking was lower, varying among the comparison groups from 21.1% to 22.1% (*p* > 0.05).

**Figure 2:**
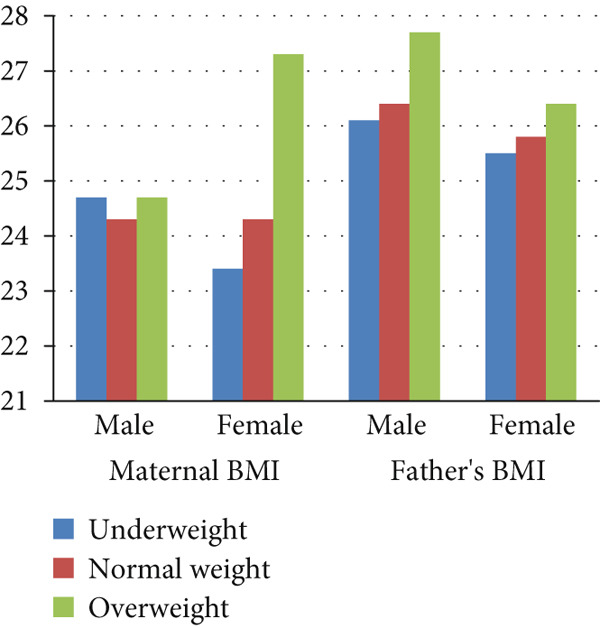
Average BMI of parents in the groups of underweight, normal weight, and overweight adolescents, kg/m^2^.

The indicators characterizing the parents′ level of education, housing conditions, and income level in the comparison groups did not feature any statistically significant differences for boys and girls (Table 6). Our comparison group is the normal body weight group. Significant differences were found in the mean values of maternal BMI for overweight girls compared with girls with normal weight (*p* < 0.05). In the other categories of adolescents with different BMI values, parents′ average BMI values did not differ significantly (Figure 2). We found that BMI of adolescents was significantly related to the maternal BMI (Pearson′s coefficient *r* = 0.225; *p* < 0.05).

**Table 6:** Family factor characteristics, abs (%).

**Indicator**	**Indicator value**	**Male**			**Female**		
		**Underweight**	**Normal weight**	**Overweight**	**Underweight**	**Normal weight**	**Overweight**
Mother′s education	Higher	18 (29.5%)	76 (28.6%)	18 (31.6%)	22 (40.7%)	93 (35.1%)	28 (37.3%)
	Secondary special	36 (59.0%)	150 (56.4%)	34 (59.6%)	29 (53.7%)	146 (55.1%)	39 (52.0%)
	Secondary general	6 (9.8%)	39 (14.7%)	5 (8.8%)	3 (5.6%)	25 (9.4%)	8 (10.7%)
	Incomplete secondary	1 (1.6%)	1 (0.4%)	0 (0.0%)	0 (0.0%)	1 (0.4%)	0 (0.0%)

Father′s education	Higher	11 (20.4%)	52 (21.2%)	10 (18.2%)	15 (31.3%)	65 (27.0%)	26∗ (38.8%)
	Secondary special	32 (59.3%)	151 (61.6%)	40 (72.7%)	29 (60.4%)	147 (61.0%)	34 (50.7%)
	Secondary general	10 (18.5%)	40 (16.3%)	4 (7.3%)	4 (8.3%)	25 (10.4%)	7 (10.4%)
	Incomplete secondary	1 (1.9%)	2 (0.8%)	1 (1.8%)	0 (0.0%)	4 (1.7%)	0 (0.0%)

Adolescents′ perceptions of family income	Lower than others	8 (12.5%)	22 (8.0%)	5 (8.1%)	6 (10.5%)	26 (9.5%)	7 (9.1%)
	Same as others	53 (82.8%)	231 (84.0%)	53 (85.5%)	47 (82.5%)	233 (85.0%)	66 (85.7%)
	Higher than others	3 (4.7%)	22 (8.0%)	4 (6.5%)	4 (7.0%)	15 (5.5%)	4 (5.2%)
Who does the teenager live with?	With parents	50 (78.1%)	208 (75.6%)	46 (74.2%)	32 (56.1%)	172 (62.8%)	42 (54.5%)
	With other relatives	7 (10.9%)	27 (9.8%)	6 (9.7%)	4 (7.0%)	26 (9.5%)	8 (10.4%)
	With husband/wife	1 (1.6%)	6 (2.2%)	0 (0%)	1 (1.8%)	4 (1.5%)	5∗ (6.5%)
	With a friend/girlfriend	2 (3.1%)	15 (5.5%)	7 (11.3%)	15 (26.3%)	47 (17.2%)	12 (15.6%)
	One	4 (6.3%)	19 (6.9%)	3 (4.8%)	5 (8.8%)	25 (9.1%)	10 (13.0%)

Housing conditions	Separate apartment	43 (67.2%)	161 (58.5%)	37 (59.7%)	31 (54.4%)	159 (58.0%)	44 (57.1%)
	Well‐maintained house	9 (14.1%)	51 (18.5%)	14 (22.6%)	5 (8.8%)	39 (14.2%)	7 (9.1%)
	Home without amenities	1 (1.6%)	5 (1.8%)	1 (1.6%)	1 (1.8%)	0 (0.0%)	1 (1.3%)
	Campus	10 (15.6%)	48 (17.5%)	9 (14.5%)	17 (29.8%)	64 (23.4%)	20 (26.0%)
	Room in shared apartment	1 (1.6%)	10 (3.6%)	1 (1.6%)	3 (5.3%)	12 (4.4%)	5 (6.5%)

Who prepares food in the family?	Parents	54 (84.4%)	238 (86.5%)	47 (75.8%)	38 (66.7%)	194 (70.8%)	52 (67.5%)
	I myself	8 (12.5%)	24 (8.7%)	11 (17.7%)	17 (29.8%)	67 (24.5%)	20 (26.0%)
	Wife/husband	0 (0.0%)	1 (0.4%)	0 (0.0%)	0 (0.0%)	0 (0.0%)	1 (1.3%)
	Other relatives?	2 (3.1%)	12 (4.4%)	4 (6.5%)	2 (3.5%)	13 (4.7%)	4 (5.2%)

How often do teenagers cook their own food?	Constantly	6 (9.4%)	21 (7.6%)	10 (16.1%)	14 (24.6%)	54 (19.7%)	17 (22.1%)
	Periodically	17 (26.6%)	90 (32.7%)	23 (37.1%)	22 (38.6%)	119 (43.4%)	37 (48.1%)
	Rarely	34 (53.1%)	135 (49.1%)	24 (38.7%)	18 (31.6%)	87 (31.8%)	22 (28.6%)
	Never	7 (10.9%)	29 (10.5%)	5 (8.1%)	3 (5.3%)	14 (5.1%)	1 (1.3%)

^∗^ *p* < 0.05 compared with the normal body weight group.

### 3.4. Regression Analysis

To establish the relationship between adolescent BMI and nutritional and nonnutritional factors, we applied the multiple linear regression method. The analysis included the indicator that had the greatest difference in diet between the comparison groups among boys and girls, which was the nutritional value of the diets. In addition to this indicator, modeling involved values characterizing the diet patterns, PAC, parents′ BMI and educational level, and adolescents’ perception of family affluence. Since all of the constructed models were of a prognostic character, we did not interpret the coefficients preceding the variables. The nature of the relationship between adolescent BMI and each of the factors studied could be judged from what was presented above in this paper. The quality of the constructed model was characterized by the value of the coefficient of determination.

The closer its value to 1, the higher the quality of the model. The constructed model′s parameters (1–4) are given in Table 7.

**Table 7:** The role of nutritional and nonnutritional factors in BMI formation, multiple regression models.

**Model**	**Predictors**	**Regression coefficient**	**t** **value**	**p** **value**	**R** ^2^
Model 1	Amount of consumed food	0.0171	33.1	< 0.001	0.86
	Simple sugars	−0.126	−19.8	< 0.001	
	Maternal BMI	0.804	10.5	< 0.001	
	Fiber	−0.207	−6.3	< 0.001	

Model 2	Maternal BMI	1.66	48.4	< 0.001	0.90
	P:F	−12.1	−22.9	< 0.001	
	Polysaccharides	−0.08	−9.8	< 0.001	
	Fiber	0.395	6.5	< 0.001	

Model 3	Amount of consumed food	0.0102	17.4	< 0.001	0.69
	P:C	−5.76	−19.3	< 0.001	
	Fats	−0.114	−10.4	< 0.001	
	PAC	−5.35	−3.6	< 0.001	

Model 4	Amount of consumed food	0.0228	28.6	< 0.001	0.77
	Mg	−0.138	−21.0	< 0.001	
	Animal proteins	0.388	18.4	< 0.001	
	Ca:Mg	45.2	15.4	< 0.001	
	Polyunsaturated fatty acids	−0.406	−9.1	< 0.001	

Model 1: the regression model of the relationship between the BMI of young men and the indicators of nutritional value, as well as familial, social, and genetic predisposition factors.

Model 2: the regression model of the relationship between the BMI of girls and the indicators of nutritional value, familial, social, and genetic predisposition factors.

Model 3: the regression model of the relationship between the BMI of young men and the indicators of nutritional value and familial and social factors.

Model 4: the regression model of the relationship between the BMI of girls and the indicators of nutritional value and familial and social factors.

Model 1 includes the following indicators: maternal BMI, amount of food consumed during the day, the diet′s content of several nutrients that perform plastic, energy, and metabolic functions in the body (simple sugars and dietary fiber). The model has a high coefficient of determination, *R* ^2^ = 0.86 (86% of the variance of the BMI indicator is explained by dependence on the selected indicators; i.e., it describes well the actual BMI values).

**Figure 3:**
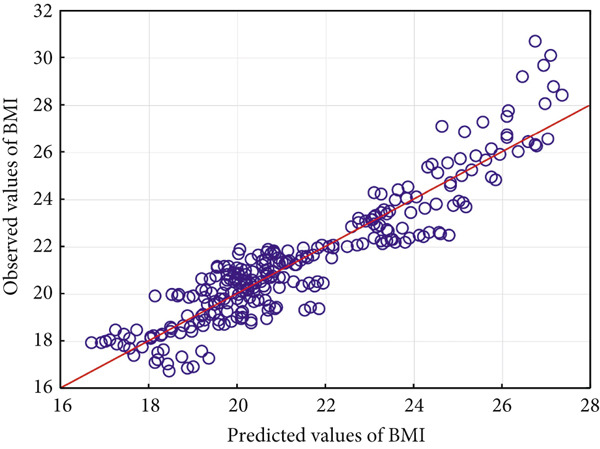
Boys′ actual/expected BMI with nutritional values and familial, social, and genetic predisposition factors.

Figure 3 shows a scatterplot of boys′ actual/expected BMI with nutritional values and familial, social, and genetic predisposition factors, which is a graphical representation of Model 1 quality.

Model 2 has an even higher coefficient of determination, *R* ^2^ = 0.90. Like Model 1, Model 2 included the indicators of maternal BMI and dietary fiber content. This model, in particular, has two diet indicators: the P:F ratio by weight, indicating how balanced the diet is regarding its fat component, and polysaccharide content. Both indicators characterize the primary sources of energy in the diet.

Among the indicators included in Models 1 and 2, the most difficult one to interpret is the maternal BMI. With reference to the time of the survey, the BMI of adolescents′ parents can be considered as a factor integrating both genetic and familial predisposition. Given the specifics of determining parental BMI, we additionally constructed two models of multiple linear regressions without taking into account the above features. The quality of the models thus constructed deteriorated a bit, though remaining quite high.

Model 3 had a fairly high coefficient of determination, *R* ^2^ = 0.69. Compared with Model 1, Model 3 has only one common indicator: the amount of food consumed. Among the new indicators included in the model, two characterized the diet (P:C ratio by weight and carbohydrate content), and one indicator characterized the PAC.

Model 4 had a high coefficient of determination, *R* ^2^ = 0.77. The list of independent features in Model 4 is completely different compared with that in Model 2. Most of the features in Model 4 characterized the state of constructive and metabolic processes: the contents of animal proteins, polyunsaturated fatty acids, and magnesium and the CA:Mg ratio in the diet. In Model 3, the amount of food consumed is also significant.

## 4. Discussion

Our results for the distribution of the BMI‐for‐age index among SVSSs in an industrial center of Russia are comparable with the sampling characteristics proposed by the WHO for constructing reference curves for the physical development of children and adolescents aged 5–19 years [2]. The findings of the study are also consistent with the ranges of prevalence data for underweight, overweight, and obese adolescents obtained in a number of studies in the Russian Federation, both multicenter ones and studies conducted in specific regions [11, 12, 42–45].

Concerning nutrition patterns, we did not find any significant differences between adolescents classified as underweight and those with normal body weight. This finding requires special further research. Probably, our study did not take into account some important underweight risk factors. It is also likely that the adolescents who were underweight at the time of the survey had recently experienced a “growth spurt” and the condition found in them was temporary. The body‐rounding period following the period of body elongation should bring their body weight to normal.

Our findings showed that overweight or obese adolescents had statistically significant differences from adolescents with normal body weight in a number of food set and nutritive value indicators. Largely, they concerned the intake of some foods or nutrients.

A recent analysis of the eating habits of 1320 adolescents from four high schools in Iaşi, Romania [46], reported gender specifics in nutrition among young people. Compared with the boys, the girls consumed fewer whole grains, dairy products, and meat, but the girls consumed fruits and vegetables in approximately the same percentage. Our findings partly support these results: gender specifics were found in nutrition among overweight young people. Overweight boys in our study differed by higher consumption of brown bread, dairy products (except for milk), eggs, and lower consumption of juices and juice‐containing products, whereas girls differed by higher consumption of meat and potatoes.

In the present study, the median caloric values of the diet among the overweight compared with those with normal body weight were statistically significantly higher only for girls. Both boys and girls with overweight differed from the corresponding comparison groups by a higher protein content of the diet, including animal proteins, as well as by the contribution of proteins to the calorie content of the diet and proportion of animal proteins in total protein content. Overweight boys featured a higher intake of saturated fatty acids and cholesterol, whereas girls consumed more simple sugars.

Our results are consistent with studies linking consumption of specific foods and food groups with health outcomes. In particular, in the present study, we found that the nutrition pattern corresponded to the “western” dietary model recognized as carrying potential risks of obesity development for boys due to high fat consumption and for girls due to high consumption of sweet foods. So, in [47], it has been pointed out that the intake of sugary beverages and regular soda during adolescence contributes toward an increase in BMI among females in early adult life. Similarly, results from the Dortmund Nutritional Anthropometric Longitudinally Designed Study showed that sugar‐sweetened beverages and regular soft drink consumption during adolescence increased BMI *Z* score by 0.07 (*p* = 0.01) and 0.1 (*p* = 0.01), respectively, in females during early adulthood. This association was not found among young males [47]. Gopinath et al. [48] found in Australia 12‐year‐old girls that each 1 SD increase in a dietary glycemic load was associated with a 0.77 kg/m^2^ increase in BMI (*p* < 0.01) 5 years later; in addition, each 1 SD increase in dietary fiber intake was associated with a 0.44 kg/m^2^ decrease (*p* < 0.02) in BMI.

Strong relationship between unhealthy westernized dietary patterns and higher BMI in adolescents was also found in the study by Gutiérrez‐Pliego et al. [17]. Oddy et al. [23] find that “western” dietary pattern (high intake of red meat, takeaway, refined foods, and confectionary) at 14 years was associated with higher energy intake and BMI at 14 years and with BMI at 17 years (all *p* < 0.05). A “healthy” dietary pattern (high in fruit, vegetables, fish, and wholegrains) was inversely associated with BMI at 17 years (*p* < 0.05). In a study by Blondin et al. [5], three types of dietary patterns were identified: prudent, western, and alcohol. Greater adherence to the prudent DP was associated with favorable anthropometric outcomes.

Our findings of less frequent meal taking by overweight adolescents are supported by a large number of similar studies. A study [18] reported that lower meal frequency and breakfast skipping were directly associated with overweight/obesity (*p* < 0.05); however, physical inactivity was not associated with higher BMI. Children′s overweight/obesity was directly associated with lower paternal education and unemployment (OR 1.30, *p* = 0.013, and OR 1.56, *p* = 0.003, respectively). Aljuraiban et al. [24] suggested that a larger number of small meals may be associated with improved diet quality and lower BMI. This may have implications for behavioral approaches to controlling the obesity epidemic.

Of particular interest is the question of a combination of being overweight with other consequences of unhealthy diet. For instance, the International Study of Macro‐/Micronutrients and Blood Pressure (INTERMAP) established that overweight adults were consuming less wholegrain food, fresh vegetables, and fruits [49]. As a result, they feature less dietary fiber, vitamin, and mineral intakes. In our study, overweight boys were found to have higher median intakes of retinol, riboflavin, calcium, magnesium, and phosphorus compared with underweight or normal weight boys. Higher sodium and phosphorus intakes were found in overweight girls. As for other vitamins and minerals, there were no statistically significant differences between groups with different BMI. Nevertheless, this issue requires additional investigation. It is important in practical terms as well. Nutritional recommendations for overweight or obese people should take into account risks of reduced micronutrient intake as a result of less food intake, primarily animal protein, saturated fatty acids, and cholesterol. It is essential to ensure that the food mix be shifted in favor of wholegrain foods, vegetables and fruit, fish, and dairy products [49].

When constructing regression models, we identified several more features that characterized their diet and nonnutritional factors. Among them are maternal BMI, amount of food consumed, fiber content, diet simple sugar content, PAC, and magnesium content and calcium‐to‐magnesium ratio for girls. Since the regression models were obtained for the entire range of BMI values studied, they may be used to predict both underweight and overweight and obesity in adolescents. The significance of these indicators as factors influencing body weight has also been shown in a number of studies [14, 25, 26, 50, 51].

What the present study adds to previous studies are the following:

1. We found similarities and differences in the diets of overweight boys and girls compared with adolescents with normal body weight.
2. It has been established that BMI is more associated with the amount of food consumed than with the calorie content of the diet, with the content of individual nutrients (proteins, including animal proteins and various types of fats and carbohydrates) and the ratios between them.
3. The importance of diet, physical activity, and maternal BMI was confirmed according to the results of regression analysis.

### 4.1. Limitations and Strengths

One of the strengths of this study is the homogeneity of the sample in terms of age composition, social status, and income and education levels. This enabled us to evaluate the influence of various factors on BMI in boys and girls in pure form (undistorted by covariates). The other strengths are the following: a wide range of variables describing the structure and nutritional value of adolescent diets and a detailed assessment of differences in the diet of underweight, normal weight, and overweight adolescents performed using the moving average method. The study presents an assessment employing a range of indicators, which provides a basis for a systemic approach to prevention work. Another strength of this study is the application of multifactorial analysis with several explanatory variables.

In interpreting the findings of this study, one should take into account some limitations. Adolescents enrolled in the study self‐reported the details of their nutrition habits independently, and thus, information about their food intake and physical activity could be biased either way.

For obvious reasons, some respondents left some questions unanswered. Thus, 4.5% of the boys and 3.4% of the girls found it difficult to answer the question about the mother′s education level; and 11.7% of the boys and 12.7% of the girls found it difficult to answer the question about the father′s level of education. Also, 16.0% of young men found it difficult to answer the question about the height and weight of their mother and 26.2% found it difficult to answer the question about those of their father. For girls, these figures were 7.4% and 19.9%, respectively. At the same time, parents′ BMI (calculated through height and weight) in our study proved to be the most difficult indicator to interpret. On the one hand, we can consider these data as indirect indicators of genetic predisposition to certain BMI in their children. On the other hand, it should be taken into account that adolescents reported the height and weight of their parents at the time of the survey rather than at birth. Over that period of time, their parents′ weight could have changed under the influence of a number of factors, including those related to their diet and physical activity. Given this, parents′ BMI at the time of the survey can be considered as a factor characterizing integratively the genetic and familial (in terms of family lifestyle) predisposition.

A certain degree of limitation rendering less obvious the diet features and nonnutritional factors identified by us in underweight and overweight adolescents was due to the cross‐sectional design of the study chosen by us, whereby we obtained information about deviations in body weight and possible related factors simultaneously. Adolescents with BMI abnormalities can make some changes in their diet or change their level of physical activity, in consequence of which the results of the study may have been distorted. The findings of this study should, of course, be verified in subsequent prospective studies.

The reason why the features of the diets and nonnutritional factors identified by us in underweight or overweight adolescents are not so obvious is likely to be the fact we obtained information on body weight deviations and possible related factors simultaneously, that is, in a cross‐sectional study. Adolescents with BMI abnormalities can make some changes in their diet or change their levels of physical activity. Also, they could misrepresent, consciously or unconsciously, details of their food habits.

We should also emphasize that the nutritional characteristics of adolescents with different BMI values were determined with reference to general nutritional problems applicable to the entire study sample. These include insufficient consumption of bakery products, vegetables, fruits, dairy products, vegetable oil, and fish and excessive consumption of pasta, cereals, sausages, and confectionery. This focus of the diets creates conditions for early onset of chronic diseases affecting the digestive, musculoskeletal, and cardiovascular systems. Another serious problem is a high frequency of tobacco use among boys.

### 4.2. Future Research

It is in our plans to conduct studies on the following:

1. The phenomenon of undernutrition in overweight and obese adolescents
2. Risks of developing underweight in adolescents
3. Eating disorders in adolescents with overweight and obesity

## 5. Conclusions

The study provides insight into potential associations between the prevalence of underweight, overweight, and obesity among SVSSs and their association with nutritional and some nonnutritional factors.

The findings of this study lay a groundwork for the development and implementation of a range of measures aimed at improving the nutrition of SVSSs, increasing their level of physical activity (including through physical education programs), improving the quality of preventive medical checks, as well as at timely detection and correction of deviations and gaps in physical development, hygienic education, and personal development among students and their parents.

Overall, the understanding of the relationship between diet and health among young adults is important for developing programs, policies, and behavior change strategies to improve quality of life and reduce diet‐related disease burden at the population level.

## Ethics Statement

The study was conducted in accordance with the Declaration of Helsinki, and the protocol was approved by the Local Ethics Committee of the South Ural State Medical University of the Ministry of Health (Protocol No. 11 dated 09.11.2013).

## Consent

Written consent was obtained from the parents and participants of the study. The participants were also ensured of the confidentiality of their data.

## Disclosure

All authors have read and agreed to the published version of the manuscript.

## Conflicts of Interest

The authors declare no conflicts of interest.

## Author Contributions

Conceptualization, G.M.N., T.A.M., and E.D.K.; data curation, S.S.D.; formal analysis, A.N.V., T.A.M., and E.D.K.; writing original draft preparation, G.M.N., S.S.D., T.A.M., and E.D.K.; writing review and editing, G.M.N., S.S.D., T.A.M., E.D.K., and S.Y.O.; project administration, E.D.K.

## Funding

This study is supported by the Ministry of Education and Science of the Russian Federation (10.13039/501100003443) (FUMN‐2024‐0002).

## Data Availability

The data that support the findings of this study are available from the corresponding author upon reasonable request.
